# EXAMINING COGNITIVE FUNCTION IN BEREAVED OLDER PARENTS: THE MEDIATING ROLE OF DEPRESSIVE SYMPTOMS

**DOI:** 10.1093/geroni/igae098.3059

**Published:** 2024-12-31

**Authors:** Stephanie Rubinstein, Elizabeth Zelinski

**Affiliations:** University of Southern California, Los Angeles, California, United States; University of Southern California, Los Angeles, California, United States

## Abstract

Losing a child is a unique and exceptionally traumatic experience, often resulting in chronic and severe depressive symptoms. This is of increasing concern as the loss of adult children has become increasingly more common since 2010. Bereaved parents represent a psychologically vulnerable group about whom we know relatively little. In this study, we use data from a nationally representative sample of 5,265 community-dwelling older adults over age 50 from the Health and Retirement Study (HRS) to examine the relationship between child loss, depressive symptoms, and cognitive function. We hypothesize that being bereaved is significantly associated with worse cognitive scores and that this relationship will be mediated by the number of reported depressive symptoms. We conducted a cluster-adjusted path analysis on repeated cross-sectional data from 1995-2020. Controlling for sociodemographic and health factors, our findings confirmed these expectations. Both bereaved individuals and those with depressive symptoms exhibited significantly worse cognitive scores, respectively (β=-0.20, p<.001; β=-0.15, p<.001). Bereaved individuals were also significantly more likely to report depressive symptoms (β=0.09, p=.001). Depressive symptomology was found to partially mediate the relationship between bereavement and cognitive function, explaining approximately 7% of the variance. These results suggest that losing a child is associated with poorer cognitive function in older adults. This relationship is partially explained by the presence of depressive symptoms, likely associated with said loss. Our findings highlight the importance of addressing mental health concerns, such as depressive symptoms, within the context of bereavement to mitigate potential adverse effects of cognitive well-being in later life.

